# Interventions to Promote an Integrated Approach to Public Health Problems: An Application to Childhood Obesity

**DOI:** 10.1155/2012/913236

**Published:** 2012-06-26

**Authors:** Anna-Marie Hendriks, Jessica S. Gubbels, Nanne K. De Vries, Jaap C. Seidell, Stef P. J. Kremers, Maria W. J. Jansen

**Affiliations:** ^1^Academic Collaborative Centre for Public Health Limburg, Regional Public Health Service, P.O. Box 2022, 6160 HA Geleen, The Netherlands; ^2^CAPHRI School of Public Health and Primary Care, Maastricht University, P.O. Box 616, 6200 MD Maastricht, The Netherlands; ^3^Department of Health Promotion, Faculty of Health, Medicine and Life Sciences, Maastricht University, P.O. Box 616, 6200 MD Maastricht, The Netherlands; ^4^NUTRIM School for Nutrition, Toxicology and Metabolism, Maastricht University, P.O. Box 616, 6200 MD Maastricht, The Netherlands; ^5^Department of Health Sciences and the EMGO Institute for Health and Care Research, Faculty of Earth and Life Sciences, VU University Amsterdam, De Boelelaan 1085, 1081 HV Amsterdam, The Netherlands

## Abstract

Experts stress the need to bring the childhood obesity epidemic under control by means of an integrated approach. The implementation of such an approach requires the development of integrated enabling policies on public health by local governments. A prerequisite for developing such integrated public health policies is intersectoral collaboration. Since the development of integrated policies is still in its early stages, this study aimed to answer the following research question: “*What interventions can promote intersectoral collaboration and the development of integrated health policies for the prevention of childhood obesity?*” Data were collected through a literature search and observations of and interviews with stakeholders. Based on a theoretical framework, we categorized potential interventions that could optimize an integrated approach regarding children's physical activity and diet. The intervention categories included education, persuasion, incentivization, coercion, training, restriction, environmental restructuring, modeling, and enablement.

## 1. Introduction

Childhood obesity is currently considered an epidemic; prevalence rates have doubled over the last three decades. Globally, approximately 170 million children (<18 years) are estimated to be overweight or obese [1, 2], and 42 million of them are estimated to be under de age of five [3]. This rapid development has focused much attention on the problem [1–5], especially since childhood and adolescent overweight and obesity are associated with lower subjective as well as objective health [6–22], often track into adulthood [23], and consequently cause huge rises in health care costs, affecting economic growth [24–26].

 With regard to subjective health, studies among children and adolescents from 11 European countries show that overweight or obese children and adolescents have poorer scores on the dimensions of physical well-being, self-perception, social acceptance, and bullying, compared to normal weight children and adolescents [6, 8]. Another study showed that severely obese children and adolescents experience a quality of life similar to that of children and adolescents with cancer [7]. Obese children and adolescents are also more likely to seek treatment for depression [9], are more likely to be stigmatized and discriminated against [10, 11], and are more likely to have disturbed eating patterns than normal weight children and adolescents [12]. With regard to objective health, children and adolescents with overweight and obesity are more likely to suffer from metabolic syndrome (hyperinsulinemia, poor glucose tolerance, dyslipidemias, and high blood pressure) [14, 15], as well as from type-2 diabetes [16], atherosclerosis [17–19], asthma [20], nonalcoholic fatty liver disease (a chronic liver disease that may result in liver cancer) [21, 22, 27], and sleep-associated breathing disorders [28, 29]. Moreover, since childhood overweight and obesity often track into adulthood [23], the health consequences related to overweight and obesity frequently persist and culminate later in life. Obese adults are discriminated against in the context of employment, education, and healthcare; they are evaluated more negatively, and negative characteristics are attributed to them [10]. With regard to objective health, overweight and obesity in adults are associated with an increased risk of type-2 diabetes [30], cardiovascular diseases [31, 32], myocardial infarction [33], and several forms of cancer [34].

In view of these consequences and the related costs, many experts have stressed the need for governments to take action [25, 26]. Since it is recognized that health, and specifically obesity, is not only influenced by determinants within the health domain, but also by those outside this domain [2, 34, 35], experts stress the need to develop integrated solutions for this so-called “wicked problem” [36–39]. Such integrated approaches need to be developed and implemented by networks of local governments, public and private stakeholders, and health promoters [5, 39, 40].

Recent health data from New York [41] and Massachusetts [42] give rise to expectations for the efficacy of an integrated approach to childhood obesity. Experiences in France [43] and Cuba (during the so-called “Special Period”) [44] also suggest that integrated approaches are effective. Commitment to integrated approaches is formalized in so-called “Health in All Policies” (HiAP) [45], defined as *“policies in which the most relevant sectors within and outside the health domain collaborate on the aspect of health, in which the common goal is to promote or protect health”* [46]. The development of such policies requires close collaboration with other policy sectors in the early phases of development. This is referred to as “intersectoral collaboration” [46]. For most governments, intersectoral collaboration is something new, as each policy sector has so far tended to work on the basis of its own logic and without regard for the impact on other areas of society [47].

In recent years, the Dutch national government has stressed the importance of developing an integrated approach for the prevention of overweight in children [48], and, in some Dutch regions, the regional Public Health Service (PHS) has implemented training courses to stimulate intersectoral collaboration in developing such an integrated approach for local governments. Although this training course has improved knowledge among local government officials about intersectoral collaboration, outcomes in terms of actual collaboration and integrated health policies with regard to overweight prevention have been disappointing [49]. Some causes of the slow development of integrated approaches for the prevention of childhood obesity are the lack of hard scientific evidence about the effectiveness of integrated approaches [49, 50], a lack of awareness of the childhood obesity problem in sectors outside the health domain [35], heads of departments not being sufficiently involved in the development of intersectoral health policies [51], civil servants lacking the competencies to develop such policies [49], and process management being insufficiently implemented [51–53]. Additionally, some experts argue that the political climate is ambiguous; governments do not seem eager to implement restrictive or legislative policy measures since this would mean they have to confront powerful lobbies by private companies (e.g., [54–56]).

To overcome these barriers, experts stress the need for a paradigm shift in society's current way of thinking about the childhood obesity epidemic [57, 58]; childhood obesity should be regarded as a public health problem instead of an individual health problem [58]. Three factors might explain why such a paradigm shift has not yet occurred. First, the problem is not yet taken seriously outside the health sector [25, 35, 56–58]. Second, the widespread long-term effect of obesity prevention is not yet widely acknowledged, compared to the limited success of individual-based treatment [1, 59]. Third, the potential role of the physical or built environment (e.g., lack of green spaces or playgrounds) [2, 50, 59, 60] and the social environment (e.g., parenting style) (e.g., [61]) in preventing childhood overweight and obesity is not yet fully recognized [2, 59]. Thus, compared to people's individual responsibility, society and governments are not yet held, or do not yet feel, responsible for providing healthy-weight-promoting environments (also referred to as leptogenic environments) [26, 56–58, 62]. In fact, however, the effect of such environments in determining behavior is even more decisive when it comes to repetitive and automatic behaviors such as diet and physical activity [62–64]; environments that offer immediate rather than long-term benefits for healthy diet and physical activity options can be expected to result in improvements in population health [65–68]. This approach to influencing behavior is also called “nudging” [66]. Examples of nudges are designing stairs with prompts that encourage people to use them, or making fruit freely available in schools during lunch breaks. The implementation of nudges is attracting interest from governments (e.g., [48, 50, 67]) since it seems to fit in with the trend among them to be reticent about interfering in private lives of citizens [56]. Local governments can implement nudges for individuals directly by changing the physical environment, but can also indirectly stimulate organizations in their municipality to implement nudges by developing policies (e.g., by subsidizing organizations that market healthy food products for children).

In theory, the best way to design and implement environments that promote physical activity and healthy diets is by means of integrated approaches. Integrated health policies formalize such approaches, and not only enable sectors within the health domain, but also local stakeholders outside this domain to change their environment and implement health promoting nudges. Local governmental policy sectors should collaborate in order to develop such policies.

To examine which interventions might be effective in supporting local governments in the development of integrated health policies, we aimed to answer the following research question: *“What interventions can promote intersectoral collaboration and the development of integrated health policies for the prevention of childhood obesity?”*

## 2. Background

In order to provide some background information to the reflections presented in this paper, we first describe the theoretical framework used in this study and the Dutch local policy-making system.

### 2.1. Theoretical Framework

**Figure 1:**
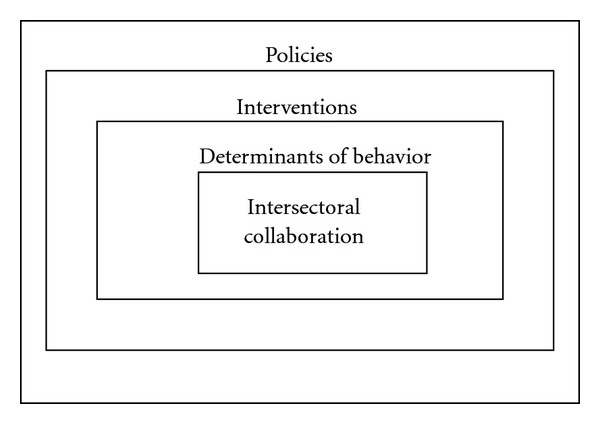
The behavior change wheel, adapted from Michie et al. [69].

We reflected on possible intervention strategies using the theoretical framework developed by Michie et al. [69] (Figure 1). We adopted this framework since it provides a clear structure for categorizing intervention functions and linking them to an analysis of the behavior of actors within the policy process. Since we aimed to explore what possible interventions might be effective, rather than finding “the best” intervention, the framework by Michie et al. [69] could help us answer our research question. The framework was originally developed for the assessment of interventions intended for the traditional target population of health promoting interventions, such as intermediaries (the people who deliver the intervention) or the ultimate target group (the children), but in this study the framework was applied to behaviors of policy makers, who are the enablers of such interventions. Intersectoral collaboration was originally developed to predict the behavior of the target population. In the original framework, intersectoral collaboration might therefore be placed in the outer circle of “policies” (e.g., as a guideline or regulation) [69]. We interpreted intersectoral collaboration as a target behavior of policy makers and therefore regard it as a behavioral goal although we recognize this interpretation might be confusing.

The framework is based on the argument that behavior is determined by the following three resources: motivation, capability, and opportunity. If one of these resources is lacking or insufficiently present, behavior change interventions might be needed to increase the likelihood of achieving a particular behavioral goal. Policies can hinder or enable the implementation of certain interventions. Intervention strategies might include education, persuasion, incentives, coercion, training, restriction, modeling, and enablement [69]. These strategies can be used to stimulate intersectoral collaboration, which should ultimately lead to integrated health policies for the prevention of overweight in children.

### 2.2. The Dutch Local Policy-Making System

Within Dutch municipal governments, three levels of actors are involved in developing integrated health policies: actors at the strategic level (the relevant alderman, the mayor, and the municipal council), the tactical level (heads of municipal departments), and the operational level (the administrative system, consisting of civil servants) [53, 70].

At the strategic level, the local political system consists of a mayor, who is not elected by local citizens but appointed by the national government, and a coalition of local politicians and aldermen who are elected every four years. This implies that the ruling political coalition can change the direction of the local policies every four years. At the tactical level, the heads of the departments manage the work processes within the administrative system. At the operational level, officials from the health department work together with the PHS to develop the public health policies [71].

The PHS is formally an extension of the health department of the municipal government, which means that the PHS staff belongs to the local government's administrative system. The degree to which the PHS is involved in policy making depends on the needs of the municipal government. Governments with larger health departments are more likely to have the capacity to draw up policy documents themselves, so their need for assistance from the PHS is usually less, while smaller municipalities often lack the capacity to develop these policies and thus delegate tasks to the PHS [71].

Avoiding administrative fragmentation and developing intersectoral policies requires vertical collaboration between each local government level as well as horizontal collaboration within these levels [45, 70]. Vertical collaboration refers to the collaboration between the relevant aldermen, the heads of municipal departments, and civil servants within one policy sector. Horizontal collaboration refers to the collaboration between the policy sectors at each level [47, 49, 53].

Since governmental agencies have a typically hierarchical organization structure, the decision to develop a new policy usually comes from one of three centralized sources at the strategic level within the municipal government: new national legislation that forces the municipal government to develop a new policy, a request from the municipal council for a new policy, or a decision on the part of the local aldermen that it is important to develop a policy [70–72].

If the initiative stems from actors at the strategic level, the “College of Mayor and Aldermen” all have to agree that it is in the interest of their municipality to prioritize a particular problem and develop policies for it. If they agree, a policy proposal is prepared by staff at the municipal Department of Public Health and PHS staff, who explore how the problem can be solved. Since multifaceted problems like the childhood obesity epidemic cannot be solved by a single sector, collaboration with other policy sectors is important at this stage [71]. In practice, however, such collaboration is hardly ever established [49].

After the (intersectoral) policy proposal has been approved by the heads of municipal departments, collectively known as the management team (MT), the College of Mayor and Aldermen receives it for a final review. When they approve the proposal, it is presented to the municipal council. The council has the final decision to accept or reject the policy proposal; they have to be convinced of the importance of investing scarce resources in a particular approach to a particular problem. If the proposal is unclear, councilors can ask the relevant alderman to explain it. The alderman may be supported in this task by civil servants of the municipal government and the PHS, who can be asked to give a presentation or work out the policy proposal in more detail [70, 71].

Since the municipal council members are democratically elected, it is important for them to examine whether the interests of the citizens are served by the policy proposal. Since council members are elected every four years, policies that result in actions that are visible to citizens within a four-year timeframe are important for council members who want to be reelected [70, 71].

## 3. Methods

Data were gathered on the basis of a framework approach [73] derived from Michie et al. [69] (Figure 1). Interviews with stakeholders, observations at meetings of the regional PHS, and reading the relevant literature were conducted iteratively. The first author went to the participants' offices to conduct the interviews and attended meetings at the PHS. The researcher was able to attend all meetings at the PHS through her job at the Academic Collaborative Centre. Since we wanted to evaluate whether the framework could be applied in a local government environment, we held interviews with participants from the three levels within the municipal government, and other important stakeholders who were familiar with the local government's situation. The interviews were recorded and had an open character. Two questions were pertinent to all interviews: *“How does political commitment for the prevention of overweight in children come about?” *and* “How does intersectoral collaboration for the prevention of overweight in children come about?*” When interesting comments were made on a particular intervention, the researcher probed the participant for more details.

The analysis involved evaluating the interviews by listening carefully to the recordings. This analysis led to the selection of relevant parts of the interviews in order to search for common concepts, and specifically for comments on interventions. The interviews were interpreted further by analyzing these relevant parts of the written transcripts of the interviews. Quotes from the interviews are used to reflect on potential interventions presented below.

Twenty interviews were held with actors at the strategic, tactical and operational levels within Dutch local governments, supplemented with personal observations at meetings of the PHS of the South Limburg region in 2011. At the strategic level, five persons were interviewed: one mayor and four aldermen and one former national politician (who had previously been an alderman). At the tactical level, one secretary of the Board of the PHS, one head of a PHS department, and one head of a municipal department were interviewed. At the operational level, 10 civil servants from the local government were interviewed, as well as two project leaders of a program that supports municipal governments throughout The Netherlands. Observations were made at 20 meetings within the PHS. In addition, the literature on intervention strategies for intersectoral collaboration was reviewed.

Our review started by exploring the basic literature recommended by experts within the field, looking for important concepts and other relevant literature. This “snowballing” search method yielded a range of important studies. Google Scholar was used to support the retrieval of the papers thus identified. When current articles could not be found through Google Scholar, we continued our search using other databases such as PubMed. In this way, additional studies and theoretical frameworks were explored. Based on the articles, thus, identified, we searched again in a range of data bases. We applied minimal exclusion criteria during our search. Some of the key concepts we looked for, but without exclusively limiting our search to them, were intersectoral collaboration, cross-sectoral collaboration, intersectoral action for health, bridging intersectoral gaps for health, interventions/tools/strategies for inter-/cross-/multi-sectoral collaboration, local governments, collaborative governance, and politics of intersectoral collaboration. This approach was expected to yield a good impression of the interventions discussed in this paper. This implies that our literature review was not exhaustive, which we considered acceptable since the aim of this study was to provide a panoramic view of possible intervention strategies, rather than a narrow literature review on specific interventions.

Furthermore, since we did not find enough literature on interventions that is specifically related to the promotion of intersectoral working methods for childhood obesity prevention within the municipal government, we also drew on literature from other scientific disciplines, such as organizational and political sciences. Finally, intervention strategies were discussed with stakeholders.

## 4. Results

This section presents the reflections based on the literature, interviews, and observations on nine possible interventions strategies to promote intersectoral collaboration and the development of an integrated approach for the prevention of childhood obesity within municipal governments.

### 4.1. Education

Education implies increasing knowledge or understanding [69]. Creating awareness through education is especially important for problems such as obesity, for which norms among the public are slowly deteriorating, and which are therefore difficult to detect. Comparing the current norms with ideal norms, or the norms in other countries or groups (e.g., high versus low socioeconomic status groups), might increase awareness and the likelihood that the problem of childhood obesity is put on the political agenda. One mayor said

*This is an important role for education. …Health is something citizens do not see. *(Mayor)**

Apart from increasing awareness, education might also be used to change the frame that dominates the discourse on a topic, since “nothing is a risk itself until it is judged to be a risk” [74], which is especially relevant for wicked problems for which the problem definition depends on ideas for solving it [36]. Actors at the strategic level might not regard themselves as being responsible for preventing childhood obesity, just as they neglected responsibility for tobacco prevention for years [75]. Education might serve as a way to point out the gains from investing in childhood obesity prevention; if a financially oriented alderman understands that prevention of childhood obesity will lead to higher work productivity later in life, he or she might become interested in investing in prevention. Ultimately, this results in cocreation by aldermen in different policy sectors. One alderman reported how education had changed his view on ways to solve the childhood obesity problem.

*Six years ago, in our previous term of office, the college of mayor and aldermen once attended a talk by a professor, who confronted us with the statement that we have created an obesogenic society. Everything is based on comfort; nobody wants to ascend a flight of stairs these days. …When you enter a public building, if you look for a lift you'll spot it immediately, but if you're looking for the stairs, it takes forever, and you'll find it tucked away in some obscure corner. …You always first have to go through a phase of raising awareness before the College takes up the issue. …It touches on so many aspects (mentions all the relevant aldermen). …So you see it's like a fan, it fans out to include the whole College. If you really want to promote health policy, it's not so much like a bundle, but more like a skewer that cuts across all policy domains. *(Alderman responsible for public health)**

However, although awareness of a particular problem might seem the most logical step toward recognizing it, actors at the strategic level often do not base their problem definition on objective analysis, but rather use it as a strategic activity to gain support for their point of view [74]. Merely educating actors at the strategic level by presenting epidemiological data can, therefore, not be expected to result in agenda setting for childhood obesity. Tailored information adapted to the portfolio of the relevant aldermen is more likely to result in effective education. However, presenting epidemiological data can boost agenda setting for those who are still unaware of the existence of the problem.

Policy sectors are not always aware of the way their policies influence health; education might increase awareness among all policy sectors, including those not involved in health [35]. An official of a municipal environmental department commented.

*There's not a great deal of knowledge about health among the local authorities. There isn't the knowledge. It's certainly not a bad idea to involve the Public Health Service. The Service could be involved from the very early stages of development. We might invite them over. Other departments are willing to do that. *(Municipal environmental department official)**

Evaluating the effects of previous policies is another way to increase awareness. However, previous attempts to create such awareness by introducing policy instruments like the Health Impact Assessment [76] have shown that awareness is not always sufficient to obtain commitment from other policy sectors.

### 4.2. Persuasion

Persuasion means that communication is used to elicit or enhance positive or negative feelings, or stimulate action [69]. Providing strategic information is intended to persuade, rather than to educate [77]. For example, informing aldermen about the possibilities for the development of the currently popular “public-private partnerships” as a way to implement policies is expected to stimulate political commitment for the prevention of overweight in children.

By whom people are persuaded depends on the social networks in which they operate [78]. Among policy actors, such networks are referred to as “policy networks” [79]. To integrate political values and beliefs, actors in the political arena need to possess political arguments and negotiation skills.

*You have to convince people and show them…why we think this is important (referring to the integrated approach to the problem of childhood obesity). *(Alderman responsible for public health and spatial planning)**

Since actors in the political arena have different interests than those at the operational level, promoting intersectoral collaboration among civil servants requires a different set of interests to be combined. This means that it is important to know how to adapt to the rationality of others. If, for example, the PHS cannot adapt to the mindset of politicians, certain health problems will not be put on the political agenda. The municipal council members and aldermen are elected every four years by individual votes and need to distinguish themselves in some way to be reelected; they need success stories based on individual health gains. The ability of the PHS to demonstrate individual progress, illustrated by narratives, is therefore expected to be more persuasive to politicians than the way the PHS currently tries to persuade them (by presenting epidemiological data). PHSs have recently recognized the following:

*On the other hand (i.e., apart from the civil servants and the Board) you're also dealing with politics. When it came to the really crucial aspects we made sure we were present at all committee meetings, all council meetings, to explain and illustrate. So in that sense we're really investing efforts in those people. …Which is often difficult, in politics it's not always the factual arguments that count. You can produce a 500-page book full of factual arguments, but that doesn't mean anything to them, it gets you nowhere. You have to be able to use the kind of arguments that count for them, and you have to know which ones they are. *(PHS official)**

Frames that dominate a discourse can be used to justify investing resources into intersectoral collaboration for the prevention of childhood obesity rather than in others problems. This is why reframing is such an important ability for actors in the policy context [80]. It implies that just as much effort should be invested in the presentation of data as in the data itself; information for politicians should be simple and sensible, while civil servants (the operational level) need more in-depth information about the causes and solutions. The politicians' task is to make the complexity of real-world problems understandable, while actors at the operational level should find out exactly what a problem entails and how it can be solved.

*So you have to look for those arguments that interest them…and that has nothing whatsoever to do with the actual health-related arguments. …If you see these people (i.e., politicians and administrative officials) often. …so that you know what they're interested in (then you know what arguments to use). *(PHS official)**

The difference between reframing and argumentation lies in the associative feelings that frames can elicit. For example, politicians sometimes claim that dietary intake and physical activity behaviors should not be controlled by government, but are the responsibility of individual citizens. Such claims are hard to counter with rational arguments since they refer to ethical rather than rational issues [81]. Additionally, heuristic arguments are more important than the quality of the argument if people have a low personal involvement with the topic at hand [82]. Hence, it is important to know how to reframe the debate [80].

### 4.3. Incentives

Incentivization means creating expectations of rewards [69], as in marketing [68].

*So personal affinity is something that, I think, if you want a political commitment, that's what you have to look for. It means too little is being invested in…In my view, in the roots of the rulers. (Social marketing aimed at politicians) Very much so. …That's marketing. …Cola…Those guys are always studying what 12-year-old kids like. That's all they ever do all day. …It's that simple. *(Former politician and alderman)**

The lack of incentives and the presence of disincentives for intersectoral collaboration within the governmental system might explain the slow development of such collaboration. A political system could also incorporate a reward system that more directly incentivizes civil servants who work across sectors, for example, by giving bonuses, or that works indirectly through creating a supportive culture.

*I think the head of a department can do a lot about this. As head of department, you can concentrate on guarding your own department's interests, and everyone will keep doing their own things, but you could also adopt a more open and positive attitude. And you can challenge your staff a bit more. You can focus on your own domain, but there are also interfaces with other domains, so you should also spend some time on that. You shouldn't just stick to your own little territory. *(Head of municipal department)**

What works as an incentive for a person depends on their values [83], so it is crucial to have a thorough understanding of the actors for whom an incentive is being developed. For example, if a municipal council member wants to be reelected (the incentive), what matters to them are votes, and this person will invest in policy topics that attract votes: a way to provide incentives. One alderman said

*Most council members are not terribly interested in health policy. It just not sexy, to use that word. It's not cool. What they're interested in is housing, spatial planning. That's what they like. Because that's what citizens ask them about. They talk about the pavement being new, or grass not being mown in time, or a tree not being pruned in time. Those are the things they notice. But health policy, that's something citizens don't see. …So it's really visibility that…What is it, what are the consequences, what's in it for me. That's the first question any council members asks. …And they are politicians, right? They're politicians. They want to score with the electorate. …They want to be reelected in four years' time, or now even in three years. Be reelected to the council. And what are citizens interested in? That tree and that pavement. Not in not being fat or such things. *(Alderman responsible for public health and spatial planning)**

Another alderman said that he had been reelected because the municipal government had invested in the prevention of childhood obesity; he had made his efforts visible by attracting media attention, which improved the town's image.

* (What you need is) a kind of motto you can link to your town. “(Town's name), a Healthy City”. That's sounds good, right? That's where you want to live, that's where you want to work, that's where you want to spend your leisure time. *(Alderman responsible for public health)**

Investments in childhood obesity prevention are expected not to diffuse quickly within municipalities because the relative advantage of investing in childhood obesity prevention is still an abstract concept to most politicians, and visible results in terms of body mass index are only observed in the long term, beyond the four-year timeframe of most politicians [70]. Thus there is a need for increased effort by the PHSs to highlight relative advantages and make health progress more visible [84].

### 4.4. Coercion

Coercion means the use of punishment in the form of penalties or disincentives [69]. One civil servant suggested that managers could coerce civil servants if they refused to collaborate with colleagues from other policy sectors, resulting in health policies that failed to become integrated.

*We sign our proposal. …Then the head of the department initials it, then the secretary initials it. …If along the way nobody looks at integration, or, in the end that's where the drive should come from. …so go back to the drawing board. …That will be uncomfortable at first, but it forces people to think. *(Civil servant at municipal health department)**

However, the expectation of coercion might also have adverse effects. Since the governmental system rewards civil servants who work without failures, most civil servants engage in risk-avoiding behaviors [85], which may suppress creativity [86]; if heads of departments express skepticism about each innovation, such as initiating intersectoral collaboration, civil servants will be unlikely to initiate it (they will experience this as a disincentive). Additionally, if innovations (e.g., new rules for working methods) are forced upon civil servants, they might be perceived as a threat, making the manager's efforts result in oral agreements which will never be implemented. A lack of congruence between the values of the managers or the organization and those of the employees might develop into what is known as a “façade of conformity” in the latter. In order to survive or succeed within the organization, employees might act as if they embrace the organization's values whereas they do not act upon them [87]. Therefore, rules are expected to be better enforced by “carrots” (promises of rewards to compliers) than by “sticks” (threats of punishment to noncompliers) [68].

### 4.5. Training

Training is intended to increase skills [69]. A recent evaluation of a training course for civil servants that was intended to increase their skills for intersectoral collaboration showed that although the course was effective in terms of increasing knowledge about integrated health policies, it did not result in more intersectoral collaboration and integrated approaches to childhood obesity in terms of concrete actions. This was attributed to the fact that the civil servants and alderman were often replaced after they had attended the course, or the civil servants involved did not have sufficient time to put the acquired skills into practice. Another cause of this poor outcome of the training could be that the skills were not “hands-on” enough and got stuck at the level of knowledge, making it look more like education. This lack of opportunity eliminated the effect of the training [49]. One alderman responsible for public health commented as follows

*Well, I think…there are enough training institutes that regard an integrated approach as their mission and that can help you. We've seen that at the PHS. To me, that was a real eye-opener. I mean, I'm not involved in health care myself, but I thought it was very, it was an eye-opener. And I think that if people get this eye-opener and then perhaps they themselves can, err, training the trainer or whatever it's called. Training the trainer? Yes, training the trainer. You could introduce that sport of thing too. But I think it's often a matter of the penny having to drop. You have to see the advantages. The gains. …You have a particular objective, for instance regarding health, like achieving a healthy weight, and you then see that you, well, you tend to think mostly about diets and dieticians and that sort of thing. But as soon as you take a broader view, a whole new world opens up. *(Alderman responsible for public health)**

Training to improve policy makers' adaptive skills is especially important in the case of childhood obesity prevention since the solutions to this “wicked” public health problem [36–38] may depend on the problem and opportunities to implement solutions in the local situation. For example, defining which stakeholders should be involved in the effort to empower parents to stimulate their children to become physically active and eat healthy food requires the municipal government or PHS to investigate which organizations are active in the environment of the individual children or their parents. This knowledge may not be available beforehand but should be proactively sought. After the stakeholders have been identified, each stakeholder should be stimulated to collaborate. This requires strategic modes of operation, which means adapting to their interests. Many experts have therefore emphasized the importance of the skill to increase the capability of leaders to persuade stakeholders (e.g., [57]). A former politician stated

* You have to convince your colleagues. Hundreds of books have been written about how to do that. You just have to master those techniques. You can read about these things. All those things have to be…It's not a matter of being right. I was convinced I was right for eight years. It's a matter of convincing others you're right. *(Former politician and alderman)**

### 4.6. Restriction

Restrictions, which may be imposed for instance by laws and regulations, are rules that define which behaviors are not allowed [69]. For example, local governments are not allowed not to allocate resources for public health issues; the Dutch public health law obliges local governments to take care of public health and develop a health policy document every four years [72]. One alderman said that this stimulated his involvement in childhood obesity prevention. Some rules are defined at national level, to prevent citizens from being subjected to a different set of policies in each municipality.

*So this municipal autonomy is sometimes a good thing as it's close to the citizens. Those municipal officials quickly realize what's going on and can take tailored measures. But the disadvantage is that you might end up with a patchwork of different measures in different municipalities. *(Mayor)**

In The Netherlands, adherence to such rules is monitored by the Health Inspectorate. They concluded that, in recent years, devolvement of tasks to local governments (which started three policy cycles ago) has yielded disappointing results [88]. So although local governments do produce health policy documents, their quality is not always sufficient.

The national government also imposes rules on civil servants and managers within the municipal government; the municipal management team (MT) is formally charged with checking whether the health policies developed by the civil servants are coordinated and integrated [51]. In everyday practice, however, the MT only checks if such rules are adhered to in terms of financial aspects. One alderman of public health referred to the following:

*You can develop a procedure on paper, but as I just said, if it remains a piece of paper it won't work. So if you…Because you do of course need to have it, because we do have a procedure here and it does work. And there's the stamp of approval from the finance department. That works. And why does it work? Because we all know that if that stamp of approval isn't there then nobody in the College of Mayor and Aldermen is willing to say yes. Right? That's very black-and-white, but that's how it is. And that's how it should be here. *(Alderman responsible for public health)**

### 4.7. Environmental Restructuring

Environmental restructuring means changing the social or physical context [69]. Social context refers to the political and public interests or the culture within an organization, while the physical context refers to the institutional design [89], the organizational structure [90], or the geographic proximity of colleagues from other policy domains. Both contexts are expected to be closely interconnected; some municipalities expect that changing the organizational structure will lead to a change in the organizational culture (e.g., [91]). For example, one municipal public health official commented that intersectoral collaboration with her colleagues from the spatial planning department was poor because they were located in another building. It is expected that when people are put together in the same space, they are naturally more inclined to discuss certain topics. Things like meetings increase physical proximity and therefore create social opportunities.

*So first of all it's important that those officials around you that you have to depend on to achieve something, that they, that they can agree with the ideas you want to realize in the end. And what I then, I'm just speaking for myself now, what I usually do is that I sound out my fellow aldermen. I think that, well, you just raise the topic of overweight prevention. How can we deal with that? And then it's a matter of making sure you're prepared, that you've thought about ways to tackle the problem. By getting round the table with the parties involved, by organizing things in spatial planning and so on. I could name a few more. *(Alderman responsible for public health)**

Political and public interests are similar to the concept of “policy stream” introduced by Kingdon [92], and the concept of “social-political context” proposed by Paulussen et al. [93]; it includes the national government and the laws and regulations it imposes, the organized political forces, civil servants at important positions, the network of organizations within the community, and other problems that prevail in the community and attract attention.

Institutional design refers to organizational structure; Mintzberg [90] distinguishes different types of structures to match different organizational purposes [94]. The theory of institutional design refers to these changes in an organization's structure as “organizational restructuring” [89]. Environmental restructuring includes changes outside the organization (e.g., the national culture or organizational climate), while organizational restructuring only refers to changes within the organization (e.g., the organizational structure). Both environments determine (indirectly) why innovations are adopted or rejected [84, 93]. For example, in some Dutch regions, the PHS has implemented a training course that aimed to increase the skills of civil servants to collaborate with other sectors. Although the civil servants adopted the new idea of working collaboratively, the organizational structure hampered the implementation of new working practices. Hence, the innovation was not implemented and did not continue after being adopted [49].

Future attempts to stimulate intersectoral collaboration may thus require organizational structures to be changed at the same time. Examples of such attempts by governments are the implementation of E-government systems [95, 96], intersectoral work teams, and matrix structures [90]. Matrix structures organize work based on a project or theme (e.g., the environments in which people live) rather than a subject (e.g., spatial planning). One municipal public health official commented as follows.

*People within our department and the other departments are not engaged in public health. However, we expect that the new department structure (referring to the new matrix structure being developed for the municipal government) will change many things. *(Public health official)**

### 4.8. Modeling

Modeling means providing an example that people can and want to copy [69]. Modeling an intended behavior change is based on classical learning theories, in which a person develops associations through observation. Bandura's [97] Social Learning Theory argues that a person becomes motivated if certain behaviors and the consequences of those behaviors are observed in a role model who is “walking the walk.” Such a model can be a person, an organization or a concept, as long as it is an example that includes an association between a cause and an effect.

A person can only be a role model if the observer can identify with him or her, or is in a similar situation. For example, after a politician has retired, he or she might still be interested in working in the political field, but in a different role, working as a role model or entrepreneur for childhood obesity prevention might be a way to stimulate others in the same situation to copy their entrepreneurship. Examples of such role models for national and local governments include First Lady Michelle Obama in the United States [5], and former politician Paul Rosenmöller in The Netherlands [98].

Organizational practices can also be used as examples to stimulate other organizations to adopt the same practices [93]. Some organizations, for example, stimulate their employees to engage in exercising, and such policies can be copied by other organizations (e.g., [99]). If a large part of the organizational network adopts a certain innovation, it increases the likelihood that others will also adopt [93].

Examples make abstract concepts, like intersectoral collaboration, more concrete and motivating.

*Yes of course you have to make it concrete. Otherwise it won't work. You just have to tackle a specific case and say, listen, this works. That's what I found, at the PHS at the time, that's what I thought was very good. They used this example…I think it's that one, with the high-rise building with those lifts and the stairs. …Yes, I found that…As soon as you hear something like that you think, wow, yeah. Anybody could have thought of that, but nobody did. *(Alderman responsible for public health and spatial planning)**

Previous research found that heads of municipal government departments reported intersectoral collaboration to be difficult to achieve because there were no concrete examples [51]. PHSs could, therefore, assist municipal authorities by providing such examples, like the program called “Youth on a Healthy Weight” (which is known in Dutch by the acronym JOGG). JOGG gathers examples and disseminates them, in a planned and systematic campaign, among their network of local governments [98].

Additionally, social interests may change through modeling; if citizens observe that children's health is improving in another but similar municipality, the municipal council might become motivated to copy the measures taken in the other municipality:

*But at another municipality they're doing a lot more, or doing less about overweight. What effect do you think that has on our citizens? The citizens see that Oud-Beijerland, our neighboring municipality, they're doing this and that about overweight. …And citizens see this and they talk to the council members and say why aren't we doing something like that? So then the council members at a certain point start to say, they're doing this and that in Oud-Beijerland, and we're doing nothing. Don't we have this problem here, mister alderman? …It's just because they see it happening elsewhere. *(Alderman responsible for public health and spatial planning)**

### 4.9. Enablement

Enablement means increasing opportunities for removing or dealing with barriers, not including training, education, or environmental restructuring [69]. Barriers to intersectoral collaboration at the strategic level might be removed by having two domains combined in one alderman's portfolio.

*So public health and spatial planning currently happen to be the responsibility of the same alderman. …And that means I can't make the link between spatial planning (and childhood obesity prevention). …But then it is mine, that's where you get integration. *(Alderman responsible for public health and spatial planning)**

A frequently mentioned barrier to implementing integrated health policies is the lack of time to manage the process. Process managers in public health often have too little time to complete tasks, or they may not be replaced if they become ill or change jobs. This barrier might be explained by the lack of involvement among heads of departments [51]. This lack of leadership at the tactical level makes the organizational culture less supportive [100], which might be a barrier to the development of integrated health policies.

*You can make your own integrated little plan (at the operational level), but it should also be targeted at (the tactical level). *(Municipal public health official)**

However, if civil servants, despite the support of their managers, do not know how to develop such policies, they could overcome this barrier by consulting experts. And, in the absence of the boundary-spanning skills of a project coordinator, an external project coordinator with such skills might be appointed. One official at a non-health-related department identified the fact that external advice is always not offered free of charge by the PHS as a barrier to involving the PHS in their policy development.

*For some reason, the PHS is not consulted…It's only when you ask a very specific question…that there are some agreements (between the municipality and the PHS) on what's included in the standard package of what the PHS is involved in within the municipality, but if it's not specific (when the question is not included in the standard package) we have to pay for it… *(Municipal environment official)**

Inflexible agreements might, therefore, represent a barrier to non-health-oriented policy sectors consulting PHSs. More flexible agreements, enabling municipal authorities to ask for health advice free of charge, might increase the involvement of the PHSs in developing non-health-related policies. Easy access to advice seems especially important to improve the collaboration with the nonhealth sectors since it often happens that a health recommendation clashes with the interest of nonhealth sectors. For example, if a spatial planning official is advised to reverse his plans in such a way that the city centre becomes less car friendly, he might expect at least some resistance from car owners. If he is also forced to pay for such advice, his motivation to ask for it is likely to decrease.

## 5. Discussion

Without governments that promote healthy nudges or restrict unhealthy ones in existing environments, the childhood obesity epidemic is expected to be difficult to bring under control [65, 66]. Integrated health policies, which are developed through intersectoral collaboration, seem to be the ideal way to design and implement sustainable environments that stimulate physical activity and healthy diets. Integrated approaches not only enable sectors within the health domain but also local stakeholders outside the health domain to change their environment [45, 46]. This may stimulate the implementation of successful interventions to promote a healthy weight. Promoting this development should be based on reflections about various interventions to promote intersectoral collaboration and the development of integrated health policies. At each level within the municipal government, a different set of interventions is expected to be most relevant. Relevance of interventions is expected to be related to the actions that need to be performed by actors at each level; strategic level actors, for example, are responsible for the decision to adopt the idea and therefore need to be persuaded rather than trained. Based on these considerations, the relevance of the various interventions is discussed below, followed by a brief discussion on the methodology of the present study.

### 5.1. Interventions Aimed at the Strategic Level: “Impossible Only Means That You Haven't Found the Solution yet”

Education, persuasion, and incentivization are probably most important interventions for actors at the strategic level; they need to be persuaded that investing in childhood obesity is urgent and receive incentives to overcome party-political and -organizational self-interests. Other strategies seem less important here since they tend to focus on capability and opportunity. These are more important at the lower levels in the municipal hierarchy. This is in line with Jansen's [53] views; strategies to increase collaboration between niches at the strategic level include brokering, sidestepping the formal system, lobbying, and agenda-setting. All these strategies can make use of persuasion and incentivization [68, 78]. Kingdon [92] also maintains that agenda setting for a new policy appears when a problem is recognized at a certain point in time (which can be achieved by increasing knowledge about the problem and by persuasion), when the way to solve the problem is accepted (which can be achieved through brokering) and when the political climate is favorable (which can be achieved by offering incentives to decision makers). However, before persuading an actor at the strategic level, they should be made more aware of the urgency of solving the problem [84]. Providing narratives that illustrate an individual's health progress is thought to be educational and persuasive to actors at the strategic level. After that, persuasion techniques are necessary to cross-boundaries or broker between the actors from the health and nonhealth sectors; they should solve the problem together. Using interpersonal channels is very important for such brokering. It makes persuasive communication more effective, which is especially important since the health and nonhealth policy sectors have a niche character [84]. Providing incentives for investing in childhood obesity prevention is, therefore, expected to compensate for the difficulty of the persuasion efforts and increase the relative advantages for those involved [84, 101]. Stakeholders, such as PHS staff, who are trying to influence the development of integrated health policies, should therefore have sufficient knowledge about what could constitute an incentive for the actors at the strategic level. Programs such as JOGG [98] seem to posses such knowledge: they proactively increase the visibility of aldermen's actions through media attention and use role models with whom actors at the strategic level can identify.

### 5.2. Interventions Aimed at the Tactical Level: “Mobilizing the Troops”

Education, training, and modeling are expected to be especially important for actors at the tactical level; these actors need to be aware of the requirements for facilitating intersectoral collaboration (e.g., making it a priority and therefore allowing time to be spent on it) and be aware that childhood obesity is a problem that is best approached in an integrated way. Furthermore, actors at the tactical level need to know how to manage the process of intersectoral collaboration to produce integrated health policies. Improving specific process management skills with regard to intersectoral collaboration is regarded as useful in this respect [52, 53]. Managers from commercial organizations or successful heads of departments from other municipal governments can educate or train actors at the tactical level. In order to have the right influence or to be opinion leaders, they need to be similar to the actors they are educating or training, but possess more skills to perform process management. Furthermore, commercial organizations may have more know-how about tools which can support the task of process management to increase intersectoral collaboration (e.g., E-government systems) [95, 96]. Learning from commercial organizations' best practices involves the intervention functions of education, training, and modeling. Modeling is expected to be particularly relevant. However, it seems that learning from others' experience is often rejected by actors at the tactical level; municipal governments seem to be rather introverted organizations. Additionally, previous research has concluded that staffing or recruitment policy is an important strategy for actors at the tactical level [53]. This can be stimulated through education and training. Heads of departments can recruit civil servants who are “team players”; recruiting individuals who are skilled and willing to collaborate will increase the likelihood that they will initiate and sustain collaboration, compared to civil servants who are incapable and unmotivated to collaborate. Besides focusing on hiring the right individuals, the tactical level also seems very important as regards developing the right organizational culture. Kotter [102], who is an expert on leading organizational change, emphasizes the need to create a change-friendly culture by creating a continuous sense of urgency to improve performance. Leaders within the organization should continuously reinforce alertness and curiosity, instead of greeting it with skepticism [86, 102].

### 5.3. Interventions Aimed at the Operational Level; “Don't Blame the Foot, If the Shoe Doesn't Fit”

Training, environmental restructuring, and enablement seemed to be particularly important for actors at the operational level; they need to be competent to work across sectors, and at the same time they depend on actors at the tactical level to allocate scarce resources, such as time. Restructuring the organizational environment, for instance, by rearranging the workspace and creating a supportive organizational culture, is usually controlled by higher-level actors. Environmental changes can stimulate actors at the operational level to initiate and sustain collaboration [102]. This is line with Steenbakkers [49] and Jansen's [53] suggestions; operational level changes can only be sustained if they are supported by higher-level changes. Blaming individuals should therefore be avoided. Steenbakkers et al. [49] also suggest that training should focus on increasing civil servants' ability to adopt a problem-based approach and to formulate concrete long-term goals. Additionally, barriers that emerge during change processes can be overcome through a proactive and creative approach by civil servants. Instead of thinking along a straight line, divergent thinking skills can enhance their ability to come up with a wider range of solutions to overcome any barriers. Increasing the available time can be achieved through prioritizing but can also be achieved by consulting the regional PHS. Moreover, PHS staff can use the experience they have gained in previous training courses to prepare civil servants to overcome previously identified barriers [49, 51].

### 5.4. Limitations

A weakness of the present study is its methodology. We chose to derive arguments from the interviews rather than from a detailed examination of certain hypotheses or cases through systematic analysis. Although we are fully aware of this limitation, we adopted this approach since we aimed to explore the field inductively first. In our further studies, we will systematically collect and analyze such data, and we want to encourage other researchers to do the same.

## 6. Conclusion

Actors within municipal governments may or may not be motivated or able to develop integrated health policies for the prevention of childhood obesity, but they are nevertheless asked by outside stakeholders to do so. Awareness of a whole range of interventions can help such stakeholders to rethink ways of stimulating or assisting municipal authorities in addressing children's physical activity and dietary habits through policy development.

Regional PHS staff can be used to persuade and incentivize actors at the strategic level by showing them success stories based on individual health gains, or by making health progress more visible within a four-year timeframe (the time to reelection of council members and aldermen). Programs that incorporate a wide range of such interventions can be tailored to the needs of the actors involved. These needs may differ depending on the stage of knowledge about the innovation [84] (e.g., the need to be educated before being persuaded) or other conditions specific to the targeted actor (e.g., the rearrangement of their workspace).

Diffusing the development of integrated health policies is assumed to start with knowledge about the topic [84]. This knowledge should be available nowadays since the Ottawa Charter already mentioned the need for integrated health policies in 1986 [103], and several training courses to stimulate their development have been implemented among municipal governments in recent years (e.g., [49]). To take the diffusion of this innovative working method a step further, interventions should be implemented that can accelerate the decision to adopt and implement the integrated approach to childhood obesity prevention by municipal governments. Hence, programs that persuade and support municipal governments should be developed and disseminated. A good example is the Dutch JOGG program [98, 104].

Future studies should examine the behavior change techniques and procedures used in programs that incorporate interventions to stimulate the development of the integrated approach to childhood obesity prevention within local government. This knowledge can be used to survey the full range of intervention options available, and to select rational options from among them.
